# Imported and Autochthonous Cases of Myiasis Caused by *Dermatobia hominis*: Taxonomic Identification Using the Internal Transcribed Spacer Region

**DOI:** 10.4269/ajtmh.18-0262

**Published:** 2018-07-30

**Authors:** Sonia Toussaint-Caire, Alejandro Woroszylski-Yoselevitz, Maria Elisa Vega-Memije, Guiehdani Villalobos, Nancy Rivas, Ricardo Alejandre-Aguilar, Mirza Romero-Valdovinos, Pablo Maravilla, Fernando Martinez-Hernandez

**Affiliations:** 1 Departamento de Dermatopatologia, Hospital General “Dr. Manuel Gea Gonzalez”, Ciudad de Mexico, Mexico;; 2 Departamento de Dermatologia y Dermato Oncologia, Hospital Angeles de Interlomas, Ciudad de Mexico, Mexico;; 3 Departamento de Ecologia de Agentes Patogenos, Hospital General “Dr. Manuel Gea Gonzalez”, Ciudad de Mexico, Mexico;; 4 Laboratorio de Entomologia, Escuela Nacional de Ciencias Biologicas, Instituto Politecnico Nacional, Ciudad de Mexico, Mexico;; 5 Departamento de Biologia Molecular e Histocompatibilidad, Hospital General “Dr. Manuel Gea Gonzalez”, Ciudad de Mexico, Mexico

## Abstract

*Dermatobia hominis* is a fly endemic to and widely distributed throughout the Americas; it is found from the southern regions of Mexico to Argentina. However, because of widespread travel, myiasis has become common in countries where neither the disease nor the species that cause this infection are endemic. Central Mexico, for instance, is not a region where myiasis is endemic. We, thus, describe three cases of *D. hominis* myiasis: two autochthonous cases from the southern part of Mexico and one imported from Costa Rica. In addition, morphological and genetic identification was performed on the maggots extracted from the patients.

## INTRODUCTION

Myiasis occurs in a great variety of vertebrate hosts, both wild and domestic, because of infestation by larvae of certain dipteran genera, such as *Dermatobia*, *Cordylobia*, *Chrysomya*, *Cuterebra*, and *Oestrus*, which feed on living tissue or body fluids.^1–4^

Different fly families distributed around the world may cause myiasis, and only some are distributed in the Americas, including *Dermatobia hominis* (Linnaeus, 1781), also known as the human botfly, berne, tórsalo, mountain worm, maggot, miruta, mucha, colmoyote, moyocuil, mosquito worm, ura, and suglacuru. *Dermatobia hominis* is one of the main causes of myiasis because of its obligate parasitism, which requires that it completes its life cycle in different vertebrates, including humans.^4,5^

*Dermatobia hominis* is distributed from Mexico through Central America and into South America.^1,3,6^ In Mexico, some autochthonous cases have been formally documented from endemic areas in the southernmost region^7,8^; however, outside these endemic areas, misidentification of *D. hominis* is common, and especially difficult to diagnose with suboptimal medical treatments.^4^

Genetic studies of myiasis-causing flies were initially performed for taxonomic identification^2^; however, molecular markers could be applied to determine the genetic variability, molecular evolution, and phylogeny of myiasis-causing flies. Few molecular studies of *D. hominis* have been performed, and few sequences are available.^4,9–12^ As with vector-borne diseases, studies of the genetic structure of arthropod populations are relevant for formulating state-level strategies to address their control and containment.

Specimens were recovered from three cases of myiasis by *D. hominis* in Mexican patients: one was imported from Costa Rica and two were autochthonous from Chiapas State, Mexico.

The patients were women who were 12 (patient 1), 18 (patient 2), and 33 (patient 3) years old; the specimens were found on the scalp, axilla, and pelvic region, respectively, where they presented as lesions with a furuncle appearance and were removed from the patients’ wounds using surgical methods. Patients 1 and 3 reported having traveled to Chiapas and patient 2 had traveled to Costa Rica. Patient 1 presented a larva of approximately 20 mm in length, patient 2 presented a larva of 10 mm in length, and patient 3 presented two larvae of 18 and 23 mm. Morphological identification was assessed, and the internal transcribed spacer-2 (ITS-2) sequence was used as a molecular marker.^11,12^

## MORPHOLOGICAL IDENTIFICATION

Maggots extracted from the patients were placed in formaldehyde for preservation and identified by their general morphology and respiratory stigmas. The larvae were dissected, and incisions were made at the cephalic and caudal segments of the specimens. These fragments were immersed in 10% potassium hydroxide to clean the exoskeleton, dehydrated in two steps with 70% and 96% alcohol, and finally treated with 5% acetic acid for 30 minutes to limit discoloration. The fixed samples were mounted on glass slides and covered with synthetic resin. Microscopic analyses were performed with a Nikon SMZ1500 stereoscopic microscope equipped with a Nikon DS-Fi1 camera (Nikon Corporation, Tokyo, Japan), and images of the cephalic and caudal segments were captured.^13,14^

**Figure 1.**
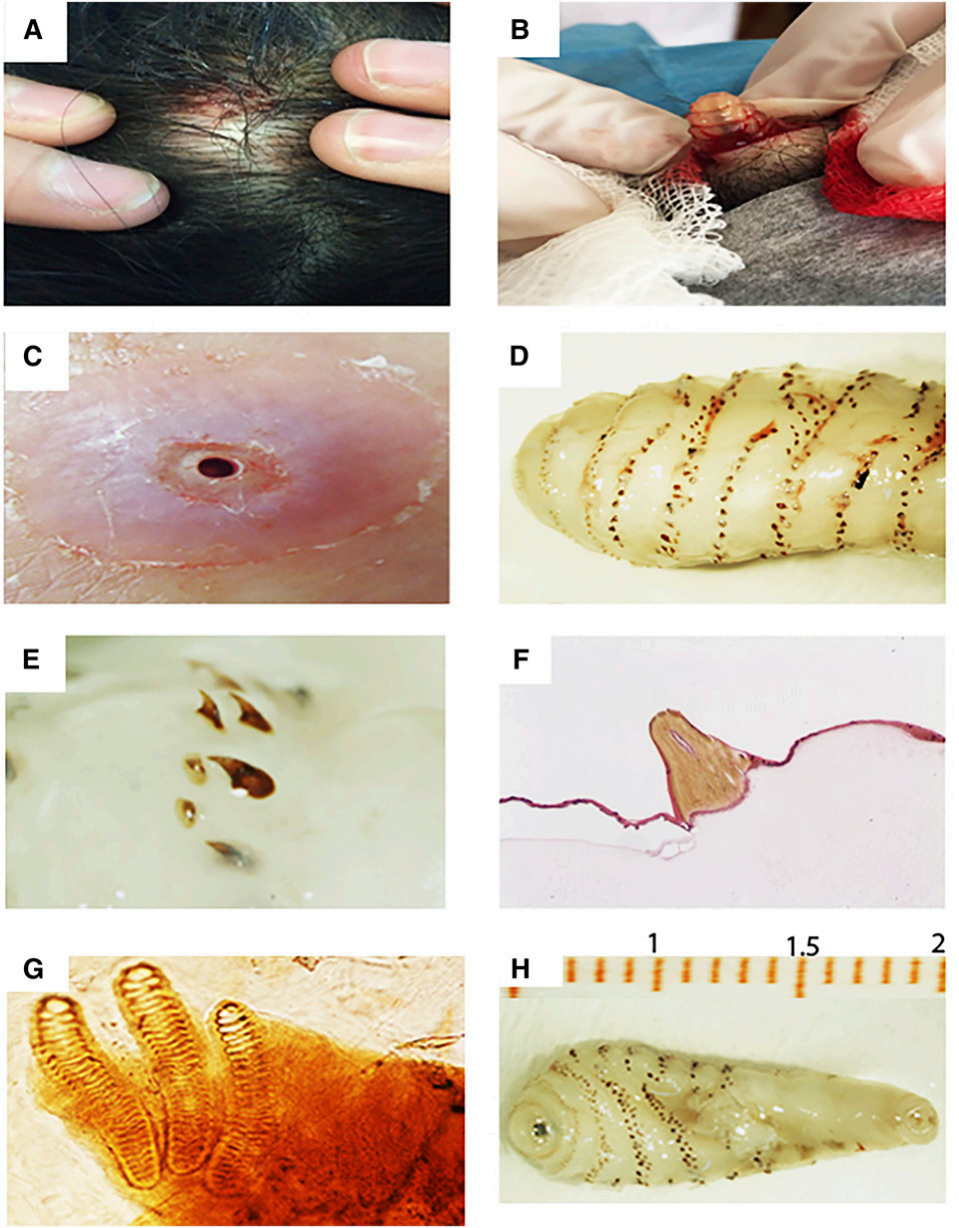
Furunculoid lesions and morphological characteristics observed in the analyzed cases: (**A**) and (**B**) lesion in head observed in case 1, (**C**) residual injuries caused by maggots observed in case 2, (**D**) body of maggots segmented by spicules, (**E**) and (**F**) spicules observed under stereoscopical and optical microscope at ×12 and ×100 magnification respectively, (**G**): second-instar larva, and (**H**) third-instar larva. This figure appears in color at www.ajtmh.org.

The maggots displayed a cylindrical shape, a yellowish-white color, and a size of 10–23 mm in length and approximately 7 mm in width (Figure 1B, D, and H). Backward-projecting spines encircling the thorax and cephalic region were observed. The four maggots were identified as *D. hominis* larvae, including one first-instar, one second-instar, and two third-instar larvae.

At the cephalic portion, where the oral opening is present, two strongly sclerotized and darkly pigmented maxilla hooks were identified. Studies of the caudal segment of the maggots showed the presence of two respiratory stigmas or respiratory spiracles that were nearly straight, with elongated anteriors and narrowed posteriors (Figure 1G), each presenting three eyelets or aperture spiracles. This anatomical structure is a species-specific characteristic of *D. hominis*. Peritreme or tegument surrounding each respiratory spiracle was not evident in any of the larvae.

**Figure 2.**
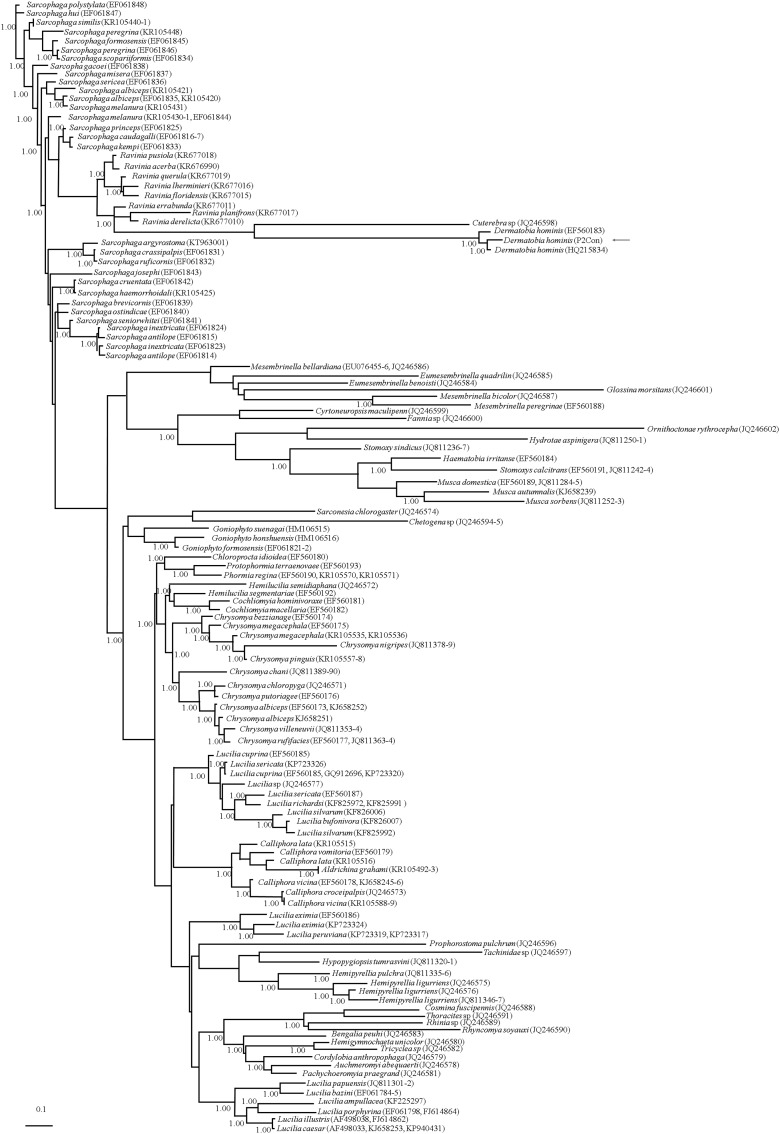
Bayesian phylogenetic tree constructed with and the internal transcribed spacer-2 sequence from Diptera species causing myiasis in the world. Numbers on branches indicate posterior probability values. Data sequences were obtained from the GenBank database; the code P2Con in *Dermatobia hominis* cluster represents those specimens obtained in the present study.

## MOLECULAR ANALYSIS

Genomic DNA was extracted from the maggots. In brief, the tissue was macerated with a tissue homogenizer (Pro200; Pro Scientific, Oxford, CT), suspended in 1 mL of lysis solution (50 mM NaCl, 10 mM Tris base, 50 mM EDTA, pH 8, 1% sodium dodecyl sulfate, and 20 μg/mL proteinase K) and incubated at 55°C overnight. The phenol–chloroform technique was used to extract DNA.^15^ The oligonucleotides used to amplify the ITS-2 sequence were reported by Marcilla et al.^16^ and the polymerase chain reaction conditions were as described previously.^17^ The sequences obtained were subjected to a BLAST search in the GenBank database and were submitted to the same database. Multiple alignments were performed using CLUSTAL W, with manual adjustment in MEGA 6.0.^18,19^ The best-fit model of nucleotide substitution was determined using the Akaike information criterion in Modeltest version 3.7^20^ and the GTR+G+I model was used. Phylogenetic reconstruction using Bayesian inference was performed with the Mr Bayes 3.1.2 program.^21^ The analysis was performed over two million generations, with sampling trees every 100 generations. Trees with scores lower than those at stationary phase were discarded. Trees that reached stationary phase were collected and used to build consensus trees. Other sequences were obtained from GenBank and used for comparison.

Little is known about ITS-2 as a molecular marker. In the phylogenetic tree, we observed that the different species of Sarcophagidae (*Sarcophaga*) and Calliphoridae (*Lucilia*) were distributed in different clades; however, the same species, regardless of genus, were grouped in specific clades. Thus, this marker is useful for classifying species but not determining phylogenetic relationships. In the case of *D. hominis* obtained in the present study, these sequences were clustered with other sequences of the same species from Brazil (HQ215834, EF560183) with differences of 17 and 13 nucleotides, corresponding to 98% identity among all sequences. In addition, this clade was grouped with the Cuterebrinae cluster (Figure 2), a subfamily of Oestridae, a grouping similar to those in other reports of fly classification using different sequences.^4,22^

## DISCUSSION

The endemic distribution of human myiasis caused by *D. hominis* extends from southern Mexico to Paraguay and northeastern Argentina; however, some cases have been reported from non-autochthonous people who travel to endemic zones.^4,23–25^ Although Mexico is an endemic country for myiasis-causing flies, only a few foreign cases have been documented.^4,26^ Here, we reported a case of myiasis acquired in Costa Rica and two cases acquired in Mexico. Interestingly, these data correlate with data published by Villalobos et al.,^4^ who commented that cases in Mexico are generally autochthonous and that Costa Rica is one of the countries with the highest number of cases in travelers. The autochthonous cases were diagnosed in a non-endemic urban area in which it is uncommon to find reports of myiasis; for these cases, adequate identification of the maggot can prevent unnecessary treatment and future complications.

In this study, we presented the morphological identification of the maggots, which exhibited structures such as spines, which develop over approximately 30–60 days depending on the time of development in the infected host^27,28^; the lack or decrease in spinulation on the terminal body segments of the larvae is a useful identification tool for differentiating *D. hominis* from other species of flies that cause myiasis.^2^ The final characterization showed maggots in the second and third stages, which are common in infected people because of the time course of the infection’s evolution. Detailed morphological identifications have rarely been described in the literature.

Few reports at the genetic level have been performed in this species of fly.^11,12^ Therefore, we conducted genetic identification using a nuclear marker, ITS-2, which has been useful for taxonomic and genetic studies in other insects.^11,12^ A total of 175 different species have been reported to cause myiasis, and this study is the first to analyze a tree constructed using the ITS-2 sequence. The gene tree showed that the ITS-2 marker clustered species of flies but was not accurate at the genus level; that is, the same genera were distributed in different branches of the tree, but similar species were grouped into the same species-specific clades. These results suggest that ITS can be used to classify species but not to establish evolutionary relationships. The individuals analyzed in this work were grouped in the clade of *Dermatobia*, next to *Cuterebra*, which is its sister genus according to other molecular markers.^9,10^

A population study of *D. hominis* using restriction fragment length polymorphism analysis of mtDNA performed in different regions of Brazil showed low genetic differentiation among populations when the diversity index was evaluated (Fst = 0.007), indicating a lack of genetic structure and that this pattern very likely reflects an intense historical gene flow or rapid expansion of the population.^10^ Interestingly, the Mexican *Dermatobia* sequences analyzed in the present study exhibited a clear difference of 2% from those of other countries, suggesting a fragmentation of the population; however, more studies are required to confirm these differences. This study is the first to describe a genetic sequence of *D. hominis* from Mexico and could contribute to further studies of the genetic variability of this important fly in the Americas.
